# Synthesis and Thermal Decomposition of High-Entropy Layered Rare Earth Hydroxychlorides

**DOI:** 10.3390/molecules29071634

**Published:** 2024-04-05

**Authors:** Maria A. Teplonogova, Anfisa A. Kozlova, Alexey D. Yapryntsev, Alexander E. Baranchikov, Vladimir K. Ivanov

**Affiliations:** 1 Kurnakov Institute of General and Inorganic Chemistry of the Russian Academy of Sciences, 119991 Moscow, Russia; 2 Faculty of Materials Science, Lomonosov Moscow State University, 119991 Moscow, Russia

**Keywords:** layered rare earth hydroxides, unit cell parameters refinement, microwave-assisted hydrothermal treatment

## Abstract

The synthesis of multicomponent and high-entropy compounds has become a rapidly developing field in advanced inorganic chemistry, making it possible to combine the properties of multiple elements in a single phase. This paper reports on the synthesis of a series of novel high-entropy layered rare earth hydroxychlorides, namely, (Sm,Eu,Gd,Y,Er)_2_(OH)_5_Cl, (Eu,Gd,Tb,Y,Er)_2_(OH)_5_Cl, (Eu,Gd,Dy,Y,Er)_2_(OH)_5_Cl, and (Eu,Gd,Y,Er,Yb)_2_(OH)_5_Cl, using a homogeneous hydrolysis technique under hydrothermal conditions. Elemental mapping proved the even distribution of rare earth elements, while luminescence spectroscopy confirmed efficient energy transfer between europium and other rare earth cations, thus providing additional evidence of the homogeneous distribution of rare earth elements within the crystal lattice. The average rare earth cation radii correlated linearly with the unit cell parameters (0.868 < R^2^ < 0.982) of the high-entropy layered rare earth hydroxychlorides. The thermal stability of the high-entropy layered rare earth hydroxychlorides was similar to that of individual hydroxychlorides and their binary solid solutions.

## 1. Introduction

Solid solutions are the most frequently used solid-state materials, and their properties can be precisely tuned by modifying their compositions. The composition of a solid solution is typically adjusted in two different ways: (1) by doping the major phase with small amounts of another component [1] or (2) by mixing almost equal amounts of several basic components. When using five or more components, the second approach leads to the formation of so-called medium- and high-entropy compounds [2]. High-entropy compounds demonstrate an even distribution of the components mixed in equal proportions, resulting in the high (>1.5 R) configurational entropy of the compound. Furthermore, high-entropy compounds may have outstanding properties in comparison with individual compounds or their binary or ternary solid solutions [3,4]. For instance, 2D layered high-entropy transition metal hydroxides show promising electrochemical catalytic activity for the oxygen evolution reaction, demonstrating a low overpotential of 275 mV at 10 mA·cm^−2^ [5].

The concept of high-entropy layered two-dimensional (2D) compounds first appeared in the 2020s and was applied to the design of novel oxides, chalcogenides, MXenes, phosphorus trichalcogenides, and hydroxides [6,7,8,9]. Among other 2D compounds, multicomponent and high-entropy layered transition metal hydroxides (layered double hydroxides) have been extensively studied in recent years [5,10,11,12,13,14,15,16]. The crystal structure of layered double hydroxides is fairly flexible, enabling convenient adjustment of both cationic and anionic compositions. For example, rare earth cation doping is a widely used method for preparing catalysts based on layered double hydroxides and further tuning their properties, such as hydrophobicity, catalytic activity, and mechanical strength [17]. Despite wide cationic flexibility, the structures of layered double hydroxides possess only limited opportunities for their doping with rare earth cations [13]. Thus, unique physical and chemical properties of rare earth cations cannot always be imparted to this type of layered hydroxides. Nevertheless, layered hydroxides can be synthesised solely from rare earth cations; (these compounds are generally referred to as layered rare earth hydroxides) [18,19]. There is, however, a limited amount of literature available on cation variation and interaction in layered rare earth hydroxides, and their structural characteristics and properties remain poorly understood. More specifically, even the correlation between the unit cell parameters and the composition of high-entropy layered hydroxides and their derivatives still have not been explored. For instance, the refinement of unit cell parameters in layered rare earth hydroxynitrates is impossible because of the stochastic coordination modes of the nitrate anions in the interlayer space [20].

Of note, other metal hydroxide-based compounds were recently synthesised in high-entropy states, for instance, dawsonite [21] and transition metal (oxy)hydroxides [22]. However, to the best of our knowledge, no high-entropy rare earth hydroxychlorides have been previously reported to be synthesized. The only paper dedicated to this closely related group of compounds, namely, high-entropy layered rare earth hydroxynitrates, was published by our team in 2022 [19]. For multicomponent and high-entropy metal hydroxide materials, the most basic properties are still severely misunderstood. One of the most interesting topics is the high-entropy effect on the thermal decomposition of metal hydroxides. Such an effect is of primary importance for the inorganic chemistry of high-entropy metal compounds, and it must be taken into account for the design and synthesis of inorganic high-entropy materials including catalysts.

This paper reports on the synthesis routes and characterisation of novel high-entropy layered rare earth hydroxychlorides. The refinement of unit cell parameters enabled the establishment of a linear correlation between unit cell parameters and the average cationic radius of multicomponent layered rare earth hydroxychlorides. The even distribution of rare earth elements suggests that, depending on their cationic composition, synthesised multicomponent layered hydroxychlorides can be classified as medium- and high-entropy compounds.

## 2. Results and Discussion

### 2.1. Characterisation of the Synthesised Multicomponent layered Rare Earth Hydroxychlorides

**Figure 1:**
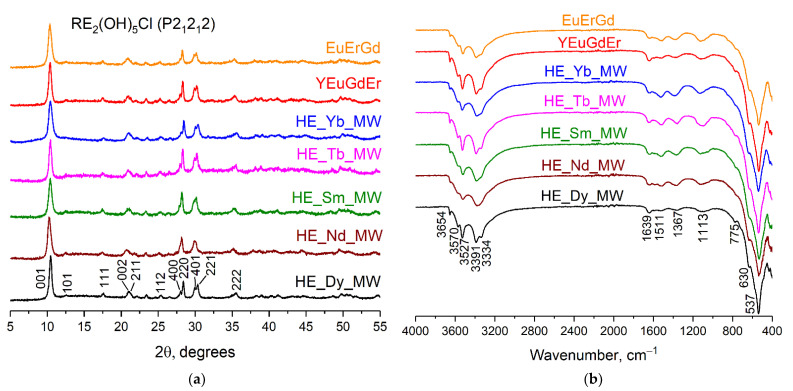
(**a**) Diffraction patterns and (**b**) FT-IR spectra of the multicomponent layered rare earth hydroxychlorides: ternary (EuErGd), quaternary (YEuErGd), and quinary (HE_RE_MW, where RE = Nd/Sm/Tb/Dy/Yb).

The XRD patterns of the synthesised samples are shown in Figure 1a. The set of diffraction reflexes corresponds to the diffraction patterns of layered rare earth hydroxychlorides, which crystallise in an orthorhombic structure (space group *P*2_1_2_1_2) [20,23]. The crystallite sizes were calculated to be 14–20 nm by applying the Scherrer equation to the diffraction maxima 001. Note that no remarkable difference was observed among the diffraction patterns of synthesised ternary, quaternary, and quinary layered rare earth hydroxychlorides.

Figure 1b shows the FT-IR spectra of the synthesised multicomponent layered rare earth hydroxychlorides. The absorption bands within the range of 3650–3330 cm^−1^ correspond to valence symmetric and antisymmetric vibrations of hydroxyl groups located in the metal-hydroxide layers. A low-intensity absorption band at 1639 cm^−1^ can be attributed to deformation vibrations of water molecules, most likely located in the interlayer space [24]. The presence of chloride ions in the samples is indicated by the bands at 1110 cm^−1^ and 630 cm^−1^ [24]. Absorption bands at 760 cm^−1^ and 540 cm^−1^ indicate vibrations of REE–O bonds [24]. Note that the FT-IR spectra contain absorption bands at 1365 cm^−1^ and 1510 cm^−1^, which indicate vibrations of carbonate ions [24,25]. The presence of carbonate anions in an interlayer space is typical for layered rare earth hydroxychlorides. In general, the FT-IR data confirm the formation of layered rare earth hydroxychlorides and show no difference among the FT-IR spectra of the ternary, quaternary, and quinary compounds.

A quantitative EDX analysis revealed that the rare earth cation ratio in the multicomponent layered hydroxychlorides corresponded to the loaded ratios. The five-cation samples HE_REE_MW had an REE ratio of about 20 at.%, the quaternary sample YEuErGd had an REE ratio of 24–26 at.%, and the ternary sample EuErGd had an REE ratio of 31–35 at.% (see Table 1). Noticeable deviations in the compositions were found only in the samples containing ytterbium and neodymium as variable cations. In both cases, the difference in the cation radii in these layered hydroxychlorides was quite significant (Table 2). This difference caused the segregation of the individual phases.

**Figure 2:**
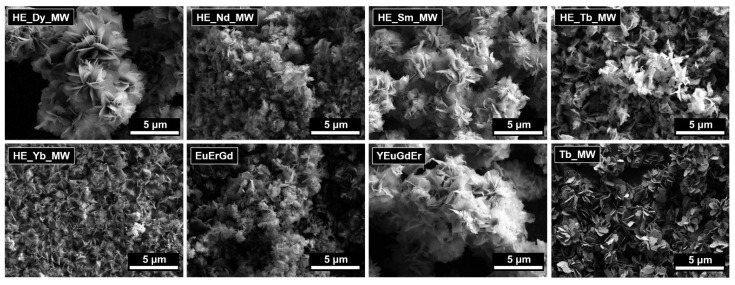
SEM images of layered terbium hydroxychloride (Tb_MW) and multicomponent layered rare earth hydroxychlorides: ternary (EuErGd), quaternary (YEuErGd), and quinary (HE_RE_MW, where RE = Nd/Sm/Tb/Dy/Yb).

The microstructures of the multicomponent layered rare earth hydroxychlorides are shown in Figure 2. These materials consisted of lamellar particles. No significant differences in the particle morphologies or sizes were found among the materials of different compositions (ternary, quaternary, and quinary layered rare earth hydroxychlorides). The lateral particle sizes measured approximately 1–3 μm, with thicknesses of about 10–20 nm. These measurements are in good agreement with the crystallite sizes calculated from the XRD data.

The lamellar shape of the particles is characteristic of layered hydroxides, particularly layered rare earth hydroxides, and is due to their layered crystal structure. The lamellar particles tend to form aggregates and their loose structure is usually observed for the layered rare earth hydroxides synthesised using microwave-assisted hydrothermal treatment at relatively low temperatures. At higher temperatures, the spherical aggregates can form via the self-assembly of individual plate-like particles [27].

### 2.2. Calculation of Configurational Entropy

Configurational entropy Δ*S*_conf_ is a crucial factor in determining whether a multicomponent compound is high-entropy or medium-entropy. To estimate Δ*S*_conf_ values for the synthesised multicomponent layered rare earth hydroxychlorides, EDX data were used (Table 3). The calculated Δ*S*_conf_ for the five-cation layered hydroxychloride was above 1.5R (with R being the universal gas constant). Therefore, the synthesised single-phase layered rare earth hydroxychlorides (HE_Sm_MW, HE_Tb_MW, HE_Dy_MW, and HE_Yb_MW) can be classified as high-entropy compounds [28,29]. The ternary (EuErGd) and the quaternary (YEuErGd) layered hydroxychlorides can be categorised as medium-entropy compounds because their configurational entropy ranged from 1R to 1.5R [28,29].

### 2.3. The Role of Cations: Unit Cell Parameters, REE Distribution, Energy Transfer, and Thermal Stability

**Figure 3:**
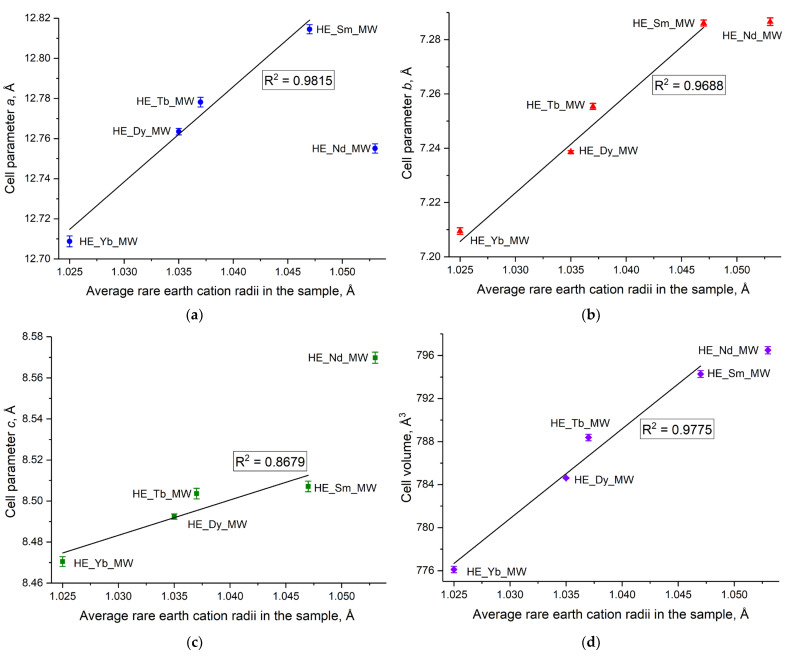
(**a**–**c**) Unit cell parameters (**a**–**d**) unit cell volume as functions of the average rare earth cation radius in high-entropy (five-component) layered rare earth hydroxychlorides HE_RE_MW (RE = Nd/Sm/Tb/Dy/Yb).

Currently, refining the unit cell parameters for layered rare earth hydroxides is only possible for certain crystal structures. For example, the crystal structure of layered rare earth hydroxynitrates is yet to be solved [20], although it is known to belong to the monoclinic structure and the space group *P*2_1_. In contrast, layered rare earth hydroxychlorides have the *P*2_1_2_1_2 space group, which belongs to the orthorhombic crystal structure, making it possible to refine the unit cell parameters for these compounds. The current study enabled the demonstration of a linear increase in unit cell parameters of high-entropy layered rare earth hydroxychlorides with an increase in the average radius of the rare earth cations in the compound (Figure 3 and Table S1 in ESI). Importantly, this increase was observed not only for parameters ***a*** and ***b***, which correspond to the distances within the metal-hydroxide layer, but also for the unit cell volume and parameter ***c***, which relates to the interlayer distance in the layered hydroxychloride. The full-profile refinement results are shown in Figure S1, in ESI. The crystal structure (space group) of layered rare earth hydroxychlorides remained intact, despite a high level of cation disorder.

The noticeable deviation from the general trend in the unit cell parameters for the sample containing Nd^3+^ may be attributed to the presence of small amounts of impurities that could not be detected by XRD analysis. The impurity phases in the HE_Nd_MW sample may have formed because of a significant difference in the radii of the rare earth cations in this sample (11.4%, see Table 2). According to the current literature, an impurity of Nd(OH)_3_ is formed during the synthesis of individual layered neodymium hydroxychloride. Additionally, Nd_2_(OH)_5_Cl·nH_2_O differed from the other layered rare earth hydroxychlorides in terms of the dependence of the interlayer distance on the relative humidity. The interlayer distance in layered neodymium hydroxychloride remained relatively high (8.55–8.58 Å) and almost constant over a wide range of relative humidities. In turn, other layered rare earth hydroxychlorides demonstrated either large (8.5–8.6 Å) or small (8.3–8.4 Å) interlayer distances with varying humidity levels [23]. The significant difference in unit cell parameters between HE_Nd_MW and the other multicomponent samples may be attributed to the formation of a highly hydrated phase during the synthesis of HE_Nd_MW.

It should be noted that synthesised single-phased high-entropy layered hydroxychloride HE_Yb_MW contains an ytterbium cation. In contrast, there is evidence from some reports that single-cation layered ytterbium hydroxychloride cannot be synthesised [23]. This fact provides evidence for the entropy-stabilisation effect in the HE_Yb_MW sample.

**Figure 4:**
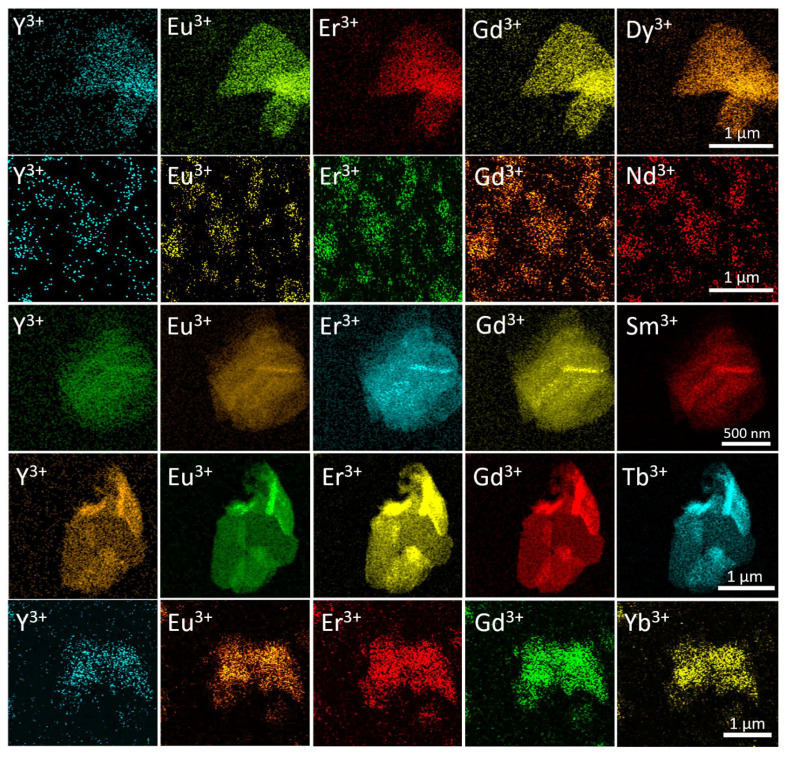
STEM-EDX elemental mapping of high-entropy layered rare earth hydroxychlorides. The elemental distributions for each sample are given in rows.

The distribution of rare earth elements in the synthesised high-entropy layered hydroxides was determined by EDX analysis with element mapping. The EDX mapping in SEM and STEM modes showed an even distribution on a submicron scale (Figure S2, ESI and Figure 4, respectively). This result is in line with the refinement of the unit cell parameters (see Figure 3).

**Figure 5:**
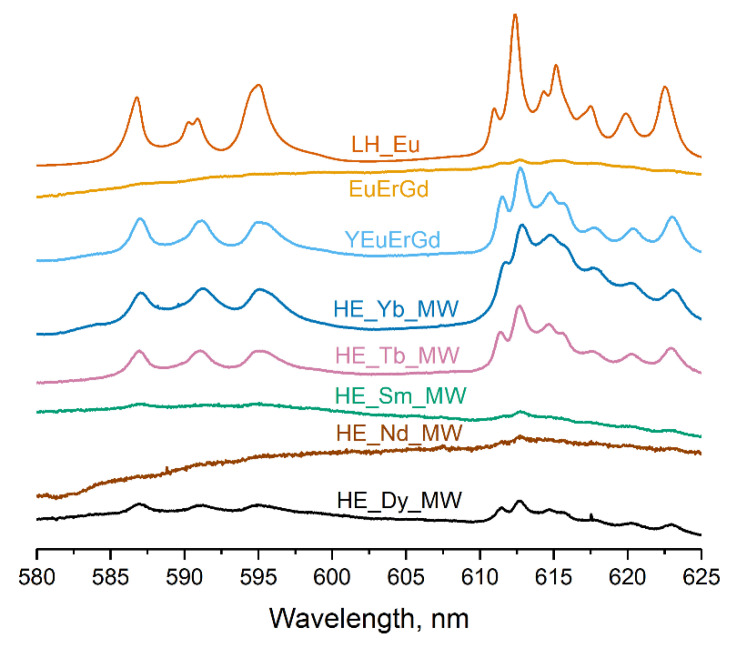
Luminescence spectra of high-entropy layered rare earth hydroxychlorides (HE_RE_MW), layered europium hydroxychloride (LH_Eu), and ternary (EuErGd) and quaternary (YEuErGd) layered rare earth hydroxychlorides.

Figure 5 presents the luminescence spectra of the multicomponent layered rare earth hydroxychlorides. All synthesised samples contained a europium cation, which re-emits electromagnetic energy in the visible region of the spectrum. The spectrum of the layered europium hydroxychloride LH_Eu shows typical luminescence bands of Eu^3+^ (580–650 nm). The ternary layered rare earth hydroxychloride (EuErGd) scarcely luminesced, probably because of efficient luminescence quenching caused by energy transfer from europium to erbium [30]. In contrast, the quaternary layered rare earth hydroxychloride (YEuErGd) showed prominent europium luminescence. This could be attributed to the addition of yttrium, which dilutes the other rare earth cations and reduces the energy transfer from Eu^3+^ to Er^3+^. As a result, europium luminescence in this compound was more intense than in the ternary layered hydroxychloride. For the high-entropy (five-cation) layered hydroxychloride, the intensity of the europium luminescence depends on the choice of the fifth variable cation. The efficient transfer of energy from the terbium cation to europium [30] resulted in stronger luminescence in the HE_Tb_MW sample. In turn, Yb^3+^ quenches the luminescence of europium, but this process is not very efficient because Yb^3+^ lacks resonance levels [30]. Therefore, the spectrum of the HE_Yb_MW sample contains well-defined luminescence bands of europium. The energy transfer from Eu^3+^ to Nd^3+^, Dy^3+^, or Sm^3+^ was highly efficient [30], leading to the quenching of europium luminescence in the HE_Nd_MW, HE_Dy_MW, and HE_Sm_MW spectra. Similar results were reported for high-entropy layered rare earth hydroxynitrates with identical cationic compositions [19]. Thus, the luminescence spectra provide evidence of energy transfer among rare earth cations in the synthesised multicomponent layered hydroxychlorides. This proves that different rare earth cations were uniformly distributed in the samples.

**Figure 6:**
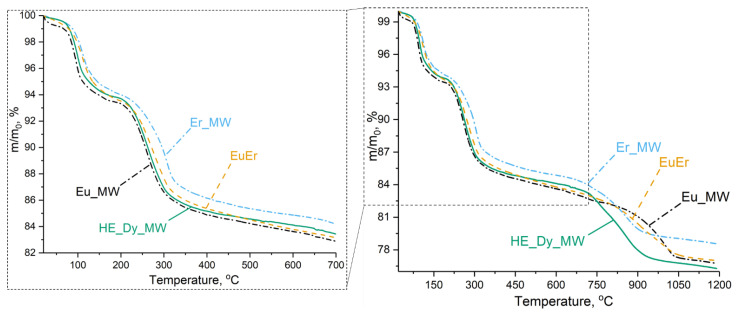
Thermal analysis results for five-cation layered hydroxychloride (HE_Dy_MW), binary layered hydroxychloride (EuEr), individual layered erbium hydroxychloride (Er_MW), and layered europium hydroxychloride (Eu_MW).

To investigate the differences in the thermal behaviours between high-entropy and low-entropy layered rare earth hydroxychlorides, a thermal analysis was carried out. The five-cation layered hydroxychloride HE_Dy_MW underwent thermal decomposition in three stages, similar to the individual and binary layered rare earth hydroxychlorides (Figure 6). Three-stage decomposition is a characteristic of layered rare earth hydroxychlorides [31]. During the first stage of the process (up to 170 °C), the layered rare earth hydroxychloride decomposes with the elimination of crystallisation water. In the second stage (170–700 °C), the layered hydroxychloride decomposes into oxide and oxychloride. The third stage of the decomposition involves the elimination of chlorine, leaving only rare earth oxides above 1050 °C.

The thermal decomposition mechanism of the high-entropy five-cation layered hydroxychloride (HE_Dy_MW) was compared with that of the layered hydroxychlorides containing the largest cation (Eu^3+^), the smallest cation (Er^3+^), and their equimolar mixture (Eu^3+^: Er^3+^ = 1: 1) (see Table 2). It is worth noting that the average cation radius in the EuEr sample was equal to that in HE_Dy_MW (1.035 Å). According to previously published data, the temperature during the third decomposition stage depends on the rare earth cation radius in an almost linear manner [31]. Specifically, a larger radius corresponds to a higher decomposition temperature (see ESI in [31]). Therefore, it was expected that the thermal stability of the HE_Dy_MW sample would be similar to the thermal stability of the EuEr sample.

The thermal analysis showed that the decomposition temperatures depended on the average cationic radii for single- and double-cation layered hydroxychlorides (Eu_MW, EuEr, and Er_MW). Below 700 °C, layered europium hydroxychloride decomposed earlier than layered erbium hydroxychloride. The thermal decomposition curve for the binary (europium-erbium) layered hydroxychloride is intermediate between the Eu_MW and Er_MW curves. Conversely, at temperatures above 700 °C, the Eu_MW sample decomposed slightly later than the Er_MW sample, while the EuEr curve remained between the Eu_MW and Er_MW curves. Meanwhile, below 700 °C, the HE_Dy_MW curve lies between the Eu_MW and EuEr curves. Above 700 °C, the HE_Dy_MW sample decomposed at approximately the same temperature as the Er_MW sample but with a more significant weight loss. This means that the high-entropy layered rare earth hydroxychloride had comparable thermal stability to the low-entropy layered rare earth hydroxychlorides. This conclusion is consistent with previous findings for 8- and 5-cation layered double hydroxides [11,16]. Therefore, the findings of the study reinforce the conclusion that high-entropy layered hydroxides cannot be classified as being entropy-stabilised [11].

From a thermodynamic perspective, the reaction entropy during decomposition, ΔS_decomp_, was not significantly affected by the cation composition of the compound. This is because S_conf_ contributed equally to both the final (LnO_1.5_), and the initial (LnOCl or Ln(OH)_2.5_Cl_0.5_), compounds. Therefore, the decomposition temperature was the same for both low- and high-entropy layered hydroxides. In contrast, during the melting process, the crystal lattice was destroyed and a liquid was formed. The contribution of S_conf_ is limited to the initial system entropy. Therefore, the difference between the ΔS_melt_ of single- and multication compounds will be substantial. Indeed, it has been demonstrated that high-entropy compounds have a higher melting temperature than low-entropy compounds, particularly for rare earth oxides [3].

## 3. Materials and Methods

The following reagents were used, as received, for the synthesis of layered rare earth hydroxides: NaCl (Chimmed, chemically pure), hexamethylenetetramine (AlfaAesar, 99+%), YCl_3_·6H_2_O (Lanhit, 99.99%), EuCl_3_·xH_2_O (Lanhit, 99.99%), GdCl_3_·xH_2_O (Lanhit, 99.99%), ErCl_3_·5H_2_O (Lanhit, 99.99%), NdCl_3_·6H_2_O (Lanhit, 99.90%), YbCl_3_·xH_2_O (Lanhit, 99.99%), TbCl_3_·xH_2_O (Lanhit, 99.90%), DyCl_3_·6H_2_O (Lanhit, 99.90%), and SmCl_3_·xH_2_O (Lanhit, 99.95%).

For the synthesis of high-entropy layered rare earth hydroxychlorides, microwave-assisted homogeneous precipitation under hydrothermal conditions was used. This technique has been shown to be rather reproducible and time-saving, as the use of microwave heating in combination with hydrothermal treatment ensures the fast formation of highly crystalline layered hydroxides with high yields [27,32]. Moreover, homogeneous hydrolysis of hexamethylenetetramine (HMT) and uniform formation of hydroxide nuclei ensures a high level of cation mixing and the formation of multication compounds. This approach have been previously used for the synthesis of layered rare earth hydroxides [27,33,34,35] and can be utilised for the synthesis of high-entropy compounds with homogeneous element distribution.

Here, layered rare earth hydroxychlorides were synthesised using microwave-assisted hydrothermal treatment, according to the procedure that showed perfect results for the synthesis of high-entropy layered rare earth hydroxynitrates [19]. First, 0.1 M solutions of rare earth chlorides were prepared in distilled water, and their exact concentrations were determined through complexometric titration. Next, 10.0 mL of 1 M NaCl solution (0.585 g) and 8.5 mL of 0.14 M HMT solution (0.196 g) were prepared in deionised water. The HMT solution was added to the NaCl solution while stirring. For each synthesis, four solutions of Y^3+^, Eu^3+^, Gd^3+^, and Er^3+^ chlorides and one solution of Nd^3+^/Sm^3+^/Yb^3+^/Tb^3+^/Dy^3+^ chloride were used. The volumes of rare earth chlorides were chosen to obtain equal molar ratios of rare earth cations (20 mol%). The molar ratio of REE^3+^: NaCl: HMT was 1:10:1.4. An excess of NaCl is necessary for the intercalation of chloride anions into the layered structure of rare earth hydroxides and to obtain a product with a stoichiometric composition [23]. The HMT concentration and temperature (140 °C) were previously tailored to ensure relatively slow hydrolysis of the rare earth cations and the formation of well-crystallised hydroxide phase [27,35]. Moreover, according to our observations, the use of lower or higher HMT concentrations results in a decrease in the hydroxide yield [27,35,36]. The reagent mixtures underwent sonication for 5 min to remove the dissolved CO_2_. For the subsequent microwave-assisted hydrothermal treatment, the total solution volume was adjusted to 30 mL, using deionised water. The solution was transferred to a Teflon autoclave (30% filling degree) and subjected to microwave-assisted hydrothermal treatment at 140 °C in a Milestone Ethos UP microwave oven. Heating was carried out for 5 min at 1800 W, and the exposure time at a given temperature was 30 min at 900 W. After the synthesis, the autoclave was cooled to room temperature. The precipitate was separated from the mother liquor by centrifugation (relative centrifugal force of 40,695× *g* for 5 min), washed with distilled water through repeated centrifugation (40,695× *g* for 5 min), and dried in an oven at 50 °C. The resulting samples were designated as HE_RE_MW, where RE represents the variable rare earth cation (Nd^3+^/Sm^3+^/Yb^3+^/Tb^3+^/Dy^3+^).

For comparison, layered europium hydroxychloride (Eu_MW), layered terbium hydroxychloride (Tb_MW), and layered erbium hydroxychloride (Er_MW) were synthesised, as well as binary (EuEr), ternary (EuErGd), and quaternary (YEuErGd) layered rare earth hydroxychlorides using a similar procedure.

Powder X-ray diffraction analysis (XRD) of the samples was carried out on a Bruker (Billerica, MA, USA) D8 Advance diffractometer (CuK_α_ radiation, λ = 1.54051 Å, Ni filter) in the range of 5–90° 2θ, with a step of 0.02° 2θ and a shutter speed of at least 0.05 sec/step. The unit cell parameters were refined by the Le Bail method, using TOPAS 4.2 software. Half-widths and the positions of the 001 reflex were estimated using Fityk software v.1.3.1 [37]. Crystallite sizes were calculated using the Scherrer equation (K = 0.9).

Scanning electron microscopy (SEM) images were taken using a Tescan Amber GMH (Brno, Czech Republic) scanning electron microscope. Images were obtained using an Everhart-Thornley SE detector at ×10,000–100,000 magnifications and at an accelerating voltage of 1–5 kV. Energy dispersive X-ray (EDX) spectra were recorded using an Ultim MAX EDS detector with a 100 mm^2^ active area (Oxford Instruments, Abingdon, Oxfordshire, GB) and at an accelerating voltage of 20 kV. EDX data were processed using Aztec 5.0 software. STEM element maps were taken at an accelerating voltage of 30 kV, using R-STEM (Tescan Amber GMH, Brno, Czech Republic) and Ultim MAX EDS (Oxford Instruments, Abingdon, Oxfordshire, GB) detectors.

The FT-IR spectra of the powders were taken using a Bruker ALPHA (Billerica, MA, USA) device in the attenuated total reflectance mode.

Thermal analysis of the samples was performed in air, using a TA Instruments SDTQ600 (New Castle, Delaware, USA) thermal analyser. The analysis was performed up to 1200 °C, in a synthetic air flow of 250 mL/min. The sample weights were 10–30 mg. The heating rate was 20 °C/min.

Luminescence spectra were recorded using a Raman microscope Confotech NR500 (SOL Instruments, Minsk, Belarus) with a 532 nm laser excitation, using 20× objective magnification (numerical aperture (NA) = 0.45) at ~2 mW laser power. The spot size was approximately 1.4–1.7 µm.

## 4. Conclusions

Single-phase high-entropy layered rare earth hydroxychlorides, namely, (Sm,Eu,Gd,Y,Er)_2_(OH)_5_Cl, (Eu,Gd,Tb,Y,Er)_2_(OH)_5_Cl, (Eu,Gd,Dy,Y,Er)_2_(OH)_5_Cl, and (Eu,Gd,Y,Er,Yb)_2_(OH)_5_Cl, and medium-entropy layered rare earth hydroxychlorides, (Eu,Gd,Er)_2_(OH)_5_Cl and (Eu,Gd,Y,Er)_2_(OH)_5_Cl, were successfully synthesised using the homogeneous hydrolysis technique under hydrothermal conditions. No remarkable difference between high- and medium-entropy layered rare earth hydroxychlorides was observed. The relationship between the average cationic radii in the layered rare earth hydroxychlorides and the unit cell parameters was found to be linear (0.868 < R^2^ < 0.982). The even distribution of rare earth cations enables the efficient energy transfer among them during the luminescence process. It was found that the thermal stability of the high-entropy layered rare earth hydroxychloride was similar to that of the low-entropy layered rare earth hydroxychloride.

In the authors’ opinion, the results of this study serve the understanding of the fundamental features of high-entropy inorganic materials. For instance, the thermal behaviour of metal hydroxides is of primary importance for the synthesis of multicomponent catalytic materials and predicting their functionality.

## Figures and Tables

**Table 1:** The content of rare earth cations in the samples of multicomponent layered hydroxychlorides according to EDX data.

Cation	Sample						
	HE_Dy_MW	HE_Nd_MW	HE_Sm_MW	HE_Tb_MW	HE_Yb_MW	YEuErGd	EuErGd
Eu^3+^	18.1	22.6	20.4	21.3	19.9	26.1	35.0
Gd^3+^	19.0	21.3	19.5	20.2	13.5	24.9	33.3
Er^3+^	20.0	22.2	19.5	19.8	21.4	23.6	31.7
Y^3+^	23.0	17.3	21.5	18.9	24.0	25.4	
Dy^3+^	19.9						
Nd^3+^		16.6					
Sm^3+^			19.1				
Tb^3+^				19.8			
Yb^3+^					21.2		
theoretical	20.0					25.0	33.3

**Table 2:** The differences between the maximum and the minimum cation radii relative to the average radius (effective ionic radii, coordination number VIII [26]): ∆r = (|R_max.cation_ − R_min.cation_|/R_average_)·100%.

Sample	R_average_, Å	∆r, %
HE_Nd_MW	1.053	11.4
HE_Sm_MW	1.047	8.6
HE_Tb_MW	1.037	6.8
HE_Dy_MW	1.035	6.8
HE_Yb_MW	1.025	8.8
YEuGdEr	1.036	6.8
EuErGd	1.043	6.7
EuEr	1.035	6.8
Eu_MW	1.07	0
Er_MW	1.00	0

**Table 3:** Configurational entropy values for the multicomponent layered rare earth hydroxychlorides.

Sample	ΔS_conf_, R
HE_Nd_MW	1.60
HE_Sm_MW	1.61
HE_Tb_MW	1.61
HE_Dy_MW	1.61
HE_Yb_MW	1.59
YEuGdEr	1.39
EuErGd	1.10

## Data Availability

Data is contained within the article or Supplementary Materials.

## Supplementary Materials

The following supporting information can be downloaded at: https://www.mdpi.com/article/10.3390/molecules29071634/s1, Table S1: Results of a full-profile refinement of XRD data for high-entropy layered rare earth hydroxychlorides HE_RE_MW (RE = Nd/Sm/Tb/Dy/Yb); Figure S1: The results of full-profile refinement of XRD data of high-entropy layered rare earth hydroxychlorides HE_RE_MW, where RE = (a) Nd, (b) Sm, (c) Tb, (d) Dy, and (e) Yb; Figure S2: EDX mapping in a SEM mode of the high- and medium-entropy layered rare earth hydroxychlorides. The elemental distributions of each sample are given in rows.
