# Supplementary material for: Synthesis and Thermal Decomposition of High-Entropy Layered Rare Earth Hydroxychlorides
Source: Molecules. 2024 Apr 5;29(7):1634. doi: 10.3390/molecules29071634 (PMC11013826; doi:10.3390/molecules29071634)
Supplement: Supplementary file 1 [file molecules-29-01634-s001.zip › molecules-2913966-supplementary.pdf]

Supplementary Materials

Synthesis and Thermal Decomposition of High-Entropy Layered Rare Earth Hydroxychlorides

Maria A. Teplonogova[1], Anfisa A. Kozlova[1], Alexey D. Yapryntsev[1], Alexander E. Baranchikov[1], Vladimir K. Ivanov[1,2*]

[1] Kurnakov Institute of General and Inorganic Chemistry of the Russian Academy of Sciences, Leninsky pr. 31, Moscow, 119991, Russia.

[2] Lomonosov Moscow State University, GSP-1, Leninskie Gory, Moscow, 119991, Russia.

E-mail: van@igic.ras.ru

Table S1. Results of a full-profile refinement of XRD data for high-entropy layered rare earth hydroxychlorides HE_RE_MW (RE = Nd / Sm / Tb / Dy / Yb).

Sample		***a***, Å	***b***, Å	***c***, Å	*V*, Å^3	R_{wp}
Average cationic radius increases ↓	HE_Yb_MW	12.7088(27)	7.2095(12)	8.4705(24)	776.11(30)	3.00
	HE_Dy_MW	12.7635(16)	7.23858(55)	8.4924(13)	784.61(6)	2.95
	HE_Tb_MW	12.7782(24)	7.2554(11)	8.5036(26)	788.38(30)	3.75
	HE_Sm_MW	12.8145(23)	7.2861(11)	8.5071(26)	794.28(31)	3.24
	HE_Nd_MW	12.7551(23)	7.2866(14)	8.5698(27)	796.48(33)	3.15

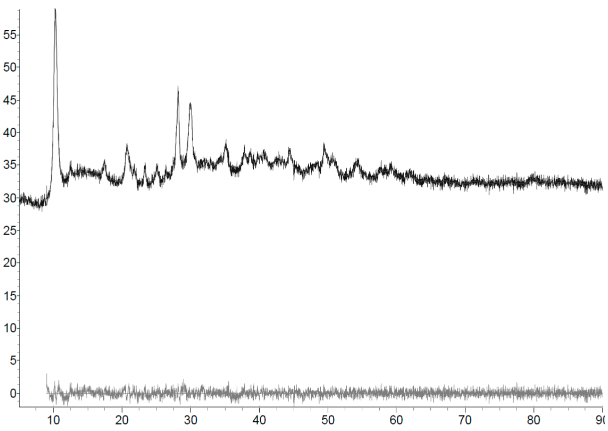

(a)

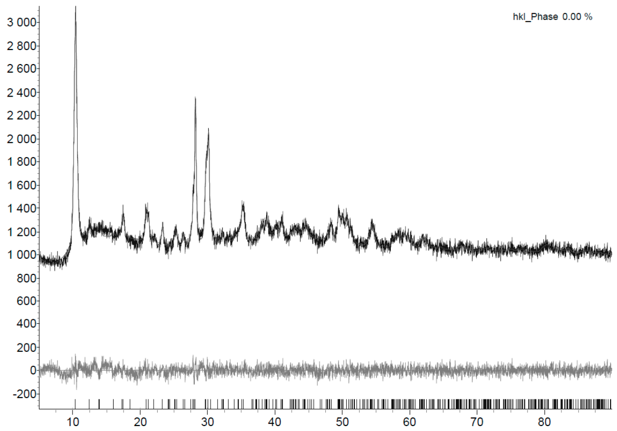

(b)

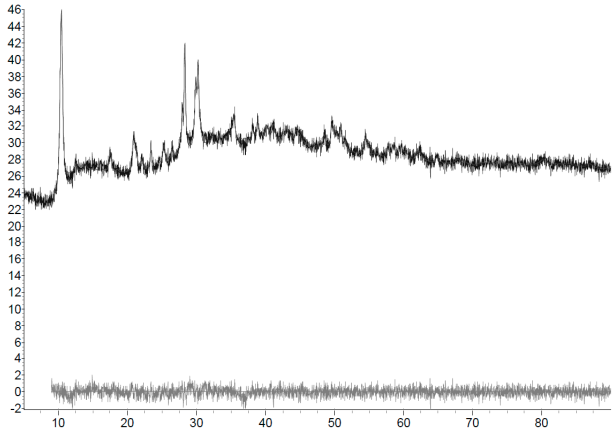

(c)

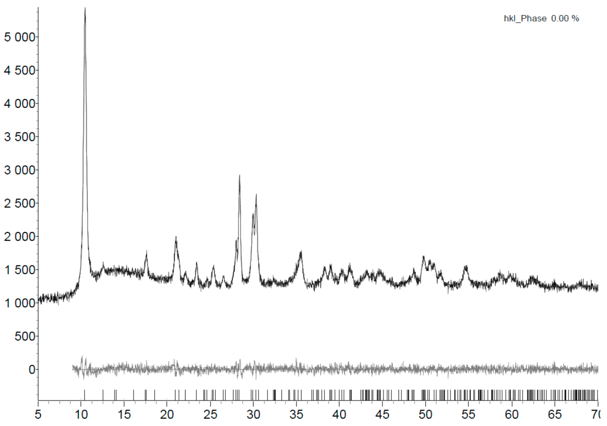

(d)

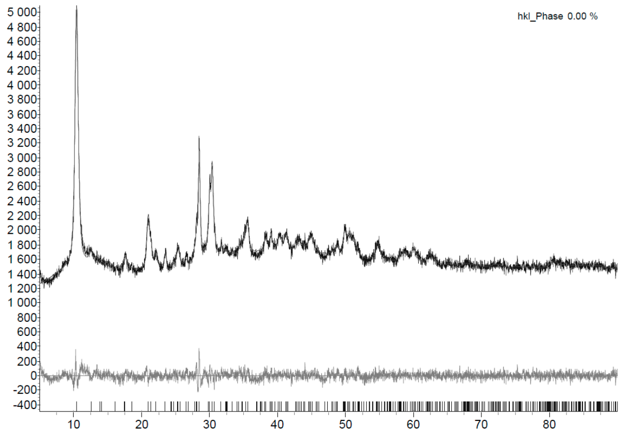

(e)

Figure S1. The results of full-profile refinement of XRD data of high-entropy layered rare earth hydroxychlorides HE_RE_MW, where RE = a) Nd, b) Sm, c) Tb, d) Dy, e) Yb.

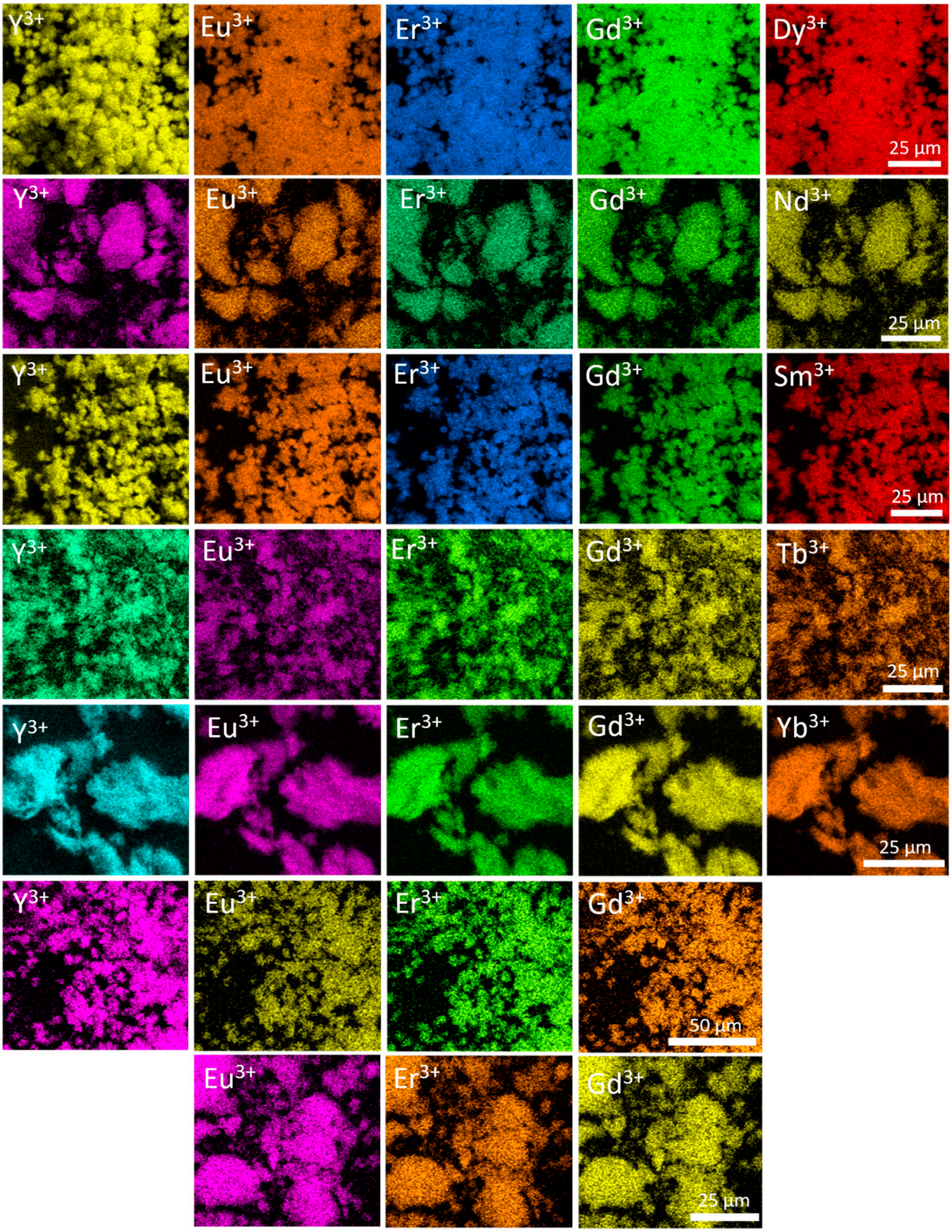

Figure S2. EDX-mapping in a SEM mode of high- and medium-entropy layered rare earth hydroxychlorides. The elemental distributions of each sample are given in rows.
